# Comparison of Carbapenemases and Extended-Spectrum β-Lactamases and Resistance Phenotypes in Hospital- and Community-Acquired Isolates of *Klebsiella pneumoniae* from Croatia

**DOI:** 10.3390/microorganisms12112224

**Published:** 2024-11-02

**Authors:** Haris Car, Mirela Dobrić, Mladen Pospišil, Marina Nađ, Josefa Luxner, Gernot Zarfel, Andrea Grisold, Ana Nikić-Hecer, Jasmina Vraneš, Branka Bedenić

**Affiliations:** 1 Zagreb Health School, 10000 Zagreb, Croatia; carharis112@gmail.com; 2 Department of Anesthesiology, Intensive Medicine and Pain Management, University Hospital Centre Sestre Milosrdnice, 10000 Zagreb, Croatia; dobric.mirela@gmail.com; 3 Department of Emergency Medicine, University Hospital Centre Zagreb, 10000 Zagreb, Croatia; mladenpospisil@gmail.com; 4 University of Zagreb School of Medicine, 10000 Zagreb, Croatia; mnad@student.mef.hr; 5 Diagnostic and Research Institute of Hygiene, Microbiology and Environmental Medicine, Medical University of Graz, 8010 Graz, Austria; josefa.luxner@medunigraz.at (J.L.); gernot.zarfel@medunigraz.at (G.Z.); andrea.grisold@medunigraz.at (A.G.); 6 Department of Microbiology and Hospital Infections, University Hospital Centre Sestre Milosrdnice, 10000 Zagreb, Croatia; ana.hecer.nikic@kbcsm.hr; 7 Department of Microbiology and Parasitology, University of Zagreb School of Medicine, Teaching Institute of Public Health “Dr. Andrija Štampar”, 10000 Zagreb, Croatia; jasmina.vranes@stampar.hr; 8 Biomedical Research Center Šalata, University of Zagreb School of Medicine, Department for Clinical Microbiology and Infection Prevention and Control, University Hospital Centre Zagreb, 10000 Zagreb, Croatia

**Keywords:** *Klebsiella pneumoniae*, OXA-48, NDM, KPC, resistance

## Abstract

*K. pneumoniae* harbors various antibiotic resistance determinants like extended-spectrum and plasmid-mediated AmpC β-lactamases and carbapenemases. In the last three years, in the period of intense population aging, migrations and climate changes in Europe and Croatia as well, we observed changes in antibiotic resistance patters of carbapenem-resistant *K. pneumoniae* (CRKP) isolates obtained routinely in community and inpatient setting. The aim was to compare and subsequently analyze CRKP hospital and community isolates resistance mechanisms, traits and molecular epidemiology, in order to analyze the dynamic of resistance trends, carbapenemase types and plasmid epidemiology. Disk diffusion and broth dilution method were the methods of choice to determine antibiotic susceptibility. β-lactamases were screened by phenotypic methods and confirmed with PCR. In total 113 isolates were analysed. Resistance to amoxicillin-clavulanate and ertapenem was confirmed in all strains. High resistance rates (over 90%) were observed for extended-spectrum cephalosporins, and ciprofloxacin. OKNV (OXA-48, KPC, NDM, VIM) testing and PCR detected OXA-48 in 106, NDM in 7 and KPC in only one isolate. ESBLs accompanied carbapenemases in 103 isolates. IncL, associated with OXA-48, was the dominant plasmid type. No significant differences in the resistance profile and resistance determinants were found between hospital and community isolates plasmid type. The predominance of OXA-48 carbapenemase is in line with the reports from the neigbouring countries.

## 1. Introduction

Of all opportunistic pathogens, *Klebsiella pneumoniae*, alongside *Acinetobacter baumannii,* is the most important due to its capability to cause severe infections like pneumonia in ventilated patients (VAPs), bloodstream infections (BSIs), urinary tract infections (UTIs), and wound infections [1,2,3]. *K. pneumoniae* isolates harbor a plethora of various antibiotic resistance determinants, including extended-spectrum β-lactamases (ESBLs), plasmid-mediated AmpC β-lactamases (p-Amp-C), and carbapenemases. Colistin is very often the last resort antibiotic, but the emergence of colistin resistance in *K. pneumoniae* limits its therapeutic use. Colistin resistance determinants are usually found in ESBL-positive and carbapenem-resistant *K. pneumoniae* (CRKP), resulting in multidrug-resistant (MDR) or extensively drug-resistant (XDR) phenotypes [4]. This poses a challenge to clinicians worldwide who treat these patients and a substantial threat to existing antibiotic armamentarium. The worldwide dissemination of CRKP and its drug resistance transfer poses a global public health threat [4].

Resistance to carbapenems in *K. pneumoniae* is mediated by two main mechanisms. The first involves the production of β-lactamases (p-AmpCs or ESBLs) with a very low level of carbapenem-hydrolyzing activity combined with decreased permeability due to porin loss or alteration. The second mechanism is attributed to carbapenem-hydrolyzing β-lactamases [5]. Carbapenemases involved in acquired resistance to carbapenems in CRKP belong to Ambler class A serin β-lactamases (KPC and GES); class B metallo-β-lactamases (MBLs) of the IMP, VIM, or NDM family; and OXA-48-like β-lactamases belonging to class D or carbapenem-hydrolyzing oxacillinases (CHDLs) [5,6,7]. The rate of CRKP in Europe is the highest in Greece, with almost 66% of isolates being carbapenem-resistant, followed by Romania (48%), Italy (29%), Bulgaria (28%), and Cyprus and Croatia (19%), respectively, according to EARS data. The Northern European countries have rates of CRKP below 0.5% [8].

Following the first report in Turkey in 2004 [9], OXA-48 spread throughout Europe, with a remarkable increase in OXA-48-producing organisms. The highest rates of OXA-48-producing organisms were observed in Turkey [9,10,11], France [12], Germany [13], Romania [14]; and in our neighboring countries Slovenia [15] and Bosnia and Herzegovina [16]. Endemic areas of OXA-48-positive *K. pneumoniae* are India, the Middle East, and North Africa, and they are very often the source of imported cases in Europe [17,18]. The gene encoding OXA-48 is plasmid-borne and located inside a composite transposon composed of two copies of the insertion sequence IS*1999* [19]. The enzyme is capable of hydrolyzing penicillins and carbapenems but has poor activity against broad-spectrum cephalosporins. Multidrug resistance in OXA-48-producing organisms often results from the coproduction of ESBLs or p-AmpC. In Italy, Greece, and Switzerland, KPC-2 and KPC-3 variants are still dominant and were associated with the pandemic clone ST258 in the past, but recently, polyclonal evolution has been reported due to the spread of high-risk lineages [20,21,22,23,24]. The most prevalent high-risk KPC-2-positive clones in Greece are ST39 and ST323, which emerged in 2019 and 2022, respectively [23]. KPC variants are typically associated with high-level resistance to all β-lactam antibiotics and in most cases to other antibiotic classes. In Switzerland, KPC positivity was linked to hypervirulence [22]. KPC carbapenemases dominate in Italian Canton [24]. MBLs belonging to the VIM and NDM family are very frequent in Greece and spread via IncA/C or IncL/M plasmid and are encoded on the genes embedded in class 1 integron [25,26] but their sporadic occurrence was also observed in Austria, usually imported from Balkan countries [27]. The new threat in Europe is the cross-border (Germany, Finland, Ireland, Italy, Slovenia, Spain, Sweden, UK, and Romania) spread of isolates harboring a combination of OXA-48 and NDM-1, exhibiting extensively drug-resistant (XDR) phenotype [14,28]. In Romania, the isolates with double carbapenememases (OXA-48+NDM) have outnumbered the isolates harboring only OXA-48 [14].

Colistin is the last resort antibiotic for the treatment of serious infections caused by CRKP. The main mechanism of colistin resistance in *K. pneumoniae* is the inactivation of the *mgr*B gene, encoding a negative feedback regulator of the *Pho*Q-*Pho*P signaling system, which activates the *pmr* system [29], responsible for the addition of positively charged phosphoethanolamine and L-arabinose to negatively charged lipid A and modifying the target for polymixins. Plasmid-mediated *mcr* genes confer resistance to colistin, transferable on mobile genetic elements [30]. Colistin resistance is frequently linked to carbapenemase production, particularly KPC-producing organisms, which were reported to cause nosocomial outbreaks in Italy [31]. Colistin-resistant carbapenemase-producing organisms pose a serious therapeutic challenge. Ceftazidime–avibactam, ceftolozane–tazobactam, imipenem–cilastatin–relebactam, and cefiderocol are the new therapeutic options for infections due to CRKP [32]. However, inhibitor combinations’ activity against MBL-positive strains is negligible; furthermore, resistance to cefiderocol has already been reported in Italy, linked to a nosocomial outbreak with NDM-1-producing *K. pneumoniae* [33].

Resistance genes in *Klebsiella* spp. are carried on conjugative plasmids. A formal scheme of plasmid classification is based on incompatibility (Inc) groups: plasmids with the same replication control are incompatible and cannot reside in the same cell line, whereas plasmids with different replication controls are compatible and can be propagated in the same cell [34].

The aim of our study was to compare the antimicrobial resistance and carbapenemase and ESBL types of hospital- and community-acquired CRKP isolates in Zagreb, Croatia, and their plasmid epidemiology in the last three years.

## 2. Materials and Method

### 2.1. Bacterial Isolates and Patients

This is a descriptive cross-section study conducted at two major hospital centers in Zagreb, Croatia: University Hospital Centre Zagreb (UHCZ) and University Hospital Centre Sestre Milosrdnice (UHCSM). The bacterial isolates included in this study were obtained during routine microbiology diagnostic. The 83 non-duplicate *K. pneumoniae* isolates with reduced susceptibility to carbapenems were collected in surgical intensive care units of the UHCZ and UHCS from 1 October 2022 to 31 December 2023 for the purpose of the study. Thirty community-acquired isolates were collected only in UHCZ in the same period. The demographic and clinical data (age, gender, underlying diseases, site of infection or colonization, and antimicrobial treatment before and after the isolation of resistant strains) were retrospectively analyzed from hospital medical records. The isolates were considered hospital-acquired if they were isolated more than 48 h after admission to the hospital. *K. pneumoniae* isolates were confirmed by MALDI-TOF MS (matrix-assisted laser desorption ionization–time-of-flight mass spectrometry) Biotyper (Bruker, Daltonik GmbH, Bremen, Germany).

### 2.2. Antimicrobial Susceptibility Testing and Phenotypic Tests for Detection of ESBLs, p-AmpC, and Carbapenemases

The initial antibiotic susceptibility testing was performed in the participating centers by the Kirby–Bauer disk diffusion test according to the EUCAST (European Committee on Antimicrobial Susceptibility Testing) guidelines [35]. Isolates exhibiting reduced susceptibility to carbapenems were subjected to further analysis. Minimum inhibitory concentrations (MICs) were determined using the broth dilution method in Mueller–Hinton broth (Oxoid, Basingstoke, UK) and 96-well microtiter plates, according to CLSI (Clinical Laboratory Standard Institution) guidelines [36] for the following antibiotics: amoxicillin–clavulanate, piperacillin–tazobactam, cefuroxime, extended-spectrum cephalosporins or ESC (ceftazidime, cefotaxime, and ceftriaxone), cefepime, imipenem, meropenem, gentamicin, amikacin, ciprofloxacin, and colistin (Sigma-Aldrich, St. Louis, MO, USA). The breakpoint of colistin was established by EUCAST; otherwise, we applied those defined by the CLSI. Minimum inhibitory concentrations (MICs) were read as the lowest concentration of an antibiotic that inhibited visible growth after 18 h at 37 °C. *E. coli* ATCC 25922 and *K. pneumoniae* 700603 were used as quality control strains for MIC determination. The susceptibility to ceftazidime–avibactam, sulphametoxazole–trimethoprim, tetracycline, and chloramphenicol was determined only using the disk diffusion test. The antibiotic-containing disks were provided by Oxoid (Basingstoke, UK). The isolates were classified as MDR, XDR, or pandrug-resistant (PDR), as described previously by Magiorakos et al. [37].

ESBL production was screened by double-disk synergy test (DDST) using amoxicillin–clavulanic acid disk opposite to ESC disks [38] and confirmed by CLSI-combined disk test using disks with ESC alone and with the addition of clavulanic acid [36]. The augmentation of the inhibition zones of cephalosporin disks of at least 5 mm by clavulanic acid confirmed ESBL production. *E. coli* ATCC 25922 and *K. pneumoniae* 700603 were used as positive and negative controls. Taking into account the susceptibility to ESC (ceftazidime, cefotaxime, and ceftriaxone) and cefepime, the isolates were assigned into three respective groups: group I (resistant to ceftazidime, cefotaxime, ceftriaxone, and cefepime), group II (resistant to ceftazidime, cefotaxime, and ceftriaxone but susceptible to cefepime), and group III (resistant to ceftazidime but susceptible to cefotaxime, ceftriaxone, and cefepime) [39].

A double-disk synergy test was performed with a disk supplemented with 500 µg cloxacillin placed between the disks containing ceftazidime and cefotaxime on a lawn of the *K. pneumoniae* isolates with reduced susceptibility to cefoxitin in order to detect p-Amp-C [40]. The distortion of the inhibition zones around ESC disks toward the central disk with cloxacillin was considered a positive result [40]. P-AmpCs were confirmed by an AmpC disk test according to Black [41]. A blank paper disk was impregnated with 20 µL Tris-EDTA to permeabilize bacterial cells. Three to five colonies of the test organism were applied to the surface of the disk. The disk was placed on the surface of MH agar previously inoculated with cefoxitin susceptible *E. coli* ATCC 25922, near the cefoxitin disk. The distortion of the inhibition zone around the cefoxitin disk indicated the enzymatic inactivation of cefoxitin [41].

The modified Hodge test (MHT) [42] and the imipenem EDTA inhibitor-based test (IDTS) [43] were performed to presumptively identify carbapenemases and MBL, respectively.

For MHT, an overnight culture of carbapenem-susceptible indicator strain *E. coli* ATCC 25922 was inoculated on the surface of MH agar plates. After drying, an ertapenem disk (10 µg) was placed in the middle of the plate. Overnight CRKP cultures were streaked as a single line from the periphery of the disc to the edge of the plate. The plates were incubated overnight at 37 °C. Carbapenemase was suspected if the clover-leaf indentation of the indicator organism was observed toward the ertapenem disc [42]. Positive control strains producing various types of carbapenemases were from our own collection.

Isolates proven to possess carbapenemase in MHT were further investigated by IDTS. Overnight CRKP culture was spread on the MH agar plate. Imipenem and meropenem disks with and without EDTA were placed on the plate. Cultures were incubated overnight at 37 °C. The augmentation of the inhibition zone around the carbapenem disk for at least 7 mm in the presence of EDTA was considered a positive result [43].

All carbapenemase-positive CRKP isolates were further tested for enzymatic hydrolysis of ertapenem using the carbapenem-inactivation method (CIM) test as described previously [44]. Positive control strains producing various types of carbapenemases were from our own collection.

Initial screening for carbapenemase types was conducted in the participating centers for the purpose of routine diagnostics with an immunochromatographic OKNV (OXA-48, KPC, NDM, and VIM) test [45].

### 2.3. Molecular Detection of Resistance Genes

Total bacterial DNA was extracted by thermal lysis. The primers and protocols used to amplify broad-spectrum and extended-spectrum β-lactamase-encoding genes (*bla*_SHV_, *bla*_TEM_, and *bla*_CTX-M_) [46,47,48] and plasmid-borne fluoroquinolone resistance genes (*qnr*A, *qnr*B, and *qnr*S) [49] were described previously. Multiplex PCR amplification was employed to identify clusters of CTX-M β-lactamase [50]; pAmpC β-lactamase genes [51]; and carbapenemase-encoding genes of class A (*bla*_KPC_) and class B (*bla*_VIM_, *bla*_IMP_, and *bla*_NDM_), CHDL (*bla*_OXA-48-like_) [52]. Plasmid-encoded colistin resistance genes *mcr*-1 and *mcr*-2 were analyzed in isolates expressing elevated MICs of colistin [30]. PCR reactions were carried out in an AC196-Alpha Cycler (PCR max, Altrincham, UK), and products were visualized by gel electrophoresis after staining with ethidium bromide.

PCR mapping was applied to analyze the genetic platform surrounding OXA-48-encoding genes, with primers for IS*1999* combined with forward and reverse primers for *bla_OXA-48_* [53]. The presence of insertion sequence preceding the *bla_CTX-M_* genes was conducted by PCR mapping with forward primer for IS*Ecp1* and IS*26* combined MA-3 primer (reverse for *bla_CTX-M_* genes) [54]. Representative amplicons were sent to Eurofin Genomics for sequencing. The positive control strains producing TEM-1, TEM-2, and SHV-1 and SHV-2 were kindly provided by Prof. Adolf Bauernfeind (Max von Pettenkofer Institute, Munich, Germany); CTX-M-15 by Prof. Neil Woodford (Health Protection Agency, London, UK); KPC-2 by Prof. Fred Tenover (Stanford University School of Medicine); and OXA-48 by Dr. Yvonne Pfeifer (Robert Koch Institute, Wernigerode, Germany).

### 2.4. Detection of Resistance Genes by the Inter-Array Kit CarbaResist

Four *K. pneumoniae* isolates were genotyped by an Inter-array chip according to the manufacturer’s recommendations (Inter-array fzmb GmbH, Bad Langensalza, Germany). The Inter-array genotyping kit CarbaResist detects broad-spectrum β-lactamases, pAmpCs, ESBLs, carbapenemases, and numerous other resistance genes (https://www.inter-array.com/further-genotyping-kits, accessed on 1 March 2024). RNA-free, unfragmented genomic DNA was isolated from the pure culture of the test strains, amplified, and internally labeled with biotin–dUDP according to the linear PCR amplification protocol using the antisense primer of the different targets only. Single-stranded DNA (ssDNA) reaction products were obtained. The biotin-labeled ssDNA was transferred to the ArrayWell and hybridized to DNA oligonucleotide microarrays with 230 probes for different β-lactam, aminoglycoside, fluoroquinolone, sulphonamide, trimethoprim, and colistin resistance genes. HRP-conjugated streptavidin was bound to the hybridized biotin-labeled ssDNA stains and visualized by enzymatic reaction. The INTER-VISION Reader was used to evaluate the spots and their intensities automatically on the basis of a digital image of the microarray. The samples obtained from the strains tested in the study were automatically analyzed for the presence or absence of specific probes and cross-checked against a database, and then information about existing resistances was output.

### 2.5. Conjugation

The conjugal transfer of ertapenem, cefotaxime, and cefoxitin resistance was carried out in mixed broth cultures at 35 °C. *E. coli* J65 resistant to sodium azide was used as recipient [55]. The ESBL- and carbapenemase-producing transconjugants were selected on MacConkey agar containing either ertapenem (0.5 mg/L) or cefotaxime (2 mg/L) and sodium azide (100 mg/L) to ensure that selected isolates were the recipient *E. coli* containing the transferred resistance gene. The frequency of conjugation was determined relative to the number of donor cells. The cotransfer of resistance to gentamicin, tetracycline, sulfamethoxazole–trimethoprim, chloramphenicol, and ciprofloxacin was determined in addition to β-lactam resistance transfer. Colonies growing on combined plates were subjected to identification by MALDI-TOF and, if confirmed to be *E. coli*, to antibiotic susceptibility testing and PCR for the detection of ESBL- and carbapenemase-encoding genes.

### 2.6. Characterization of Plasmids

The plasmid DNA of clinical isolates and their transconjugants was extracted with a Qiagen Plasmid Mini Kit (Qiagen, Hilden, Germany). After staining with ethidium bromide, the DNA was visualized by ultraviolet light.

PCR-based replicon typing (PBRT) [56] was used for molecular typing of plasmids. Eighteen pairs of primers were used, including five multiplex and three simplex PCR, in order to assess the plasmid incompatibility group. Since L/M plasmids, which usually carry *bla_OXA-48_* genes, are difficult to detect with the original protocol, an updated protocol by Carattoli was applied [34].

PCR was performed on transconjugant strains to identify if ESBL- and carbapenemase-resistant genes were transferred in the conjugation experiment. PBRT was also applied to transconjugants to identify incompatibility groups such as in their respective donors. Positive control strains for PBRT were kindly provided by Dr. A. Carattoli (Instituto Superiore di Sanita, Rome, Italy).

### 2.7. Genotyping of the ISOLATES

Clonal types of two representative *K. pneumoniae* isolates (35 and 50) were determined by multilocus sequence typing (MLST) according to Diancourt et al. [57].

### 2.8. Statistical Analysis

In order to determine whether the prevalence of ESBL-positive strains differs between hospital and community settings, we used the chi-square test of independence. The chi-square test of independence is a statistical test used to detect the presence of association between two categorical variables. The test involves the creation of a contingency table that cross-tabulates the frequencies of the two variables’ categories. The chi-square test statistic is calculated by comparing the observed and expected frequencies from the contingency table, whereas the associated *p*-value is found by comparing the test statistic to a chi-square distribution, with degrees of freedom determined by the table dimensions. A *p*-value < 0.05 was considered statistically significant.

## 3. Results

### 3.1. Bacterial Isolates and Patients

A total of 113 bacterial isolates were recovered from 113 patients and divided into three groups: UHCZ with 37 isolates, UHCSM with 46 isolates, and community setting with 30 isolates.

**Figure 1:**
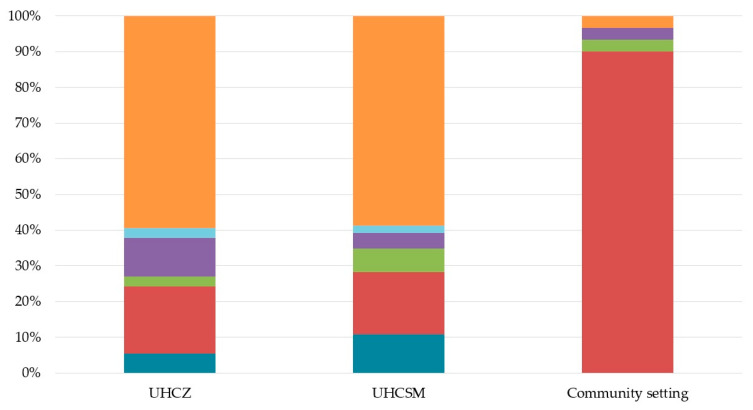
Distribution of *Klebsiella pneumoniae* isolates according to the source and sampling setting. Blood culture, blue; urine, red; wound, green; tissue, violet; BAL, light blue; surveillance culture, orange.

The age range of the patients was 2 to 95 years (mean value 67.8). This study included 68 males and 45 females. Twenty-seven patients (32%) died with a slightly higher mortality rate in UHCZ (35% vs. 30%) compared to UHCS (Supplementary Material). The urinary tract was the dominant source of CRKP (34%, *n* = 39), followed by blood cultures (9.7%, *n* = 11), tissue samples (6.2%, *n* = 7), and wounds (3.5%, *n* = 4), as shown in Figure 1. The rest of the strains originated from surveillance cultures (throat swab, rectum swab, stool, etc.), as shown in Figure 1. UHCZ’s surgical intensive care unit was the setting from which the majority of isolates were acquired (28), followed by the abdominal surgery department with 2 isolates. Other clinical departments (medical ICU, children’s surgery ward, urology department, cardiac surgery department, neurology ICU, and organ transplant unit) contributed with one isolate, respectively, as illustrated in Supplementary Materials. ICUs were the most frequent source of CRKP in the UHCSM, providing 36 isolates, while six and four isolates were recovered from the post-intensive care unit and burn unit, respectively.

### 3.2. Antimicrobial Susceptibility and Phenotypic Tests for β-Lactamases

**Figure 2:**
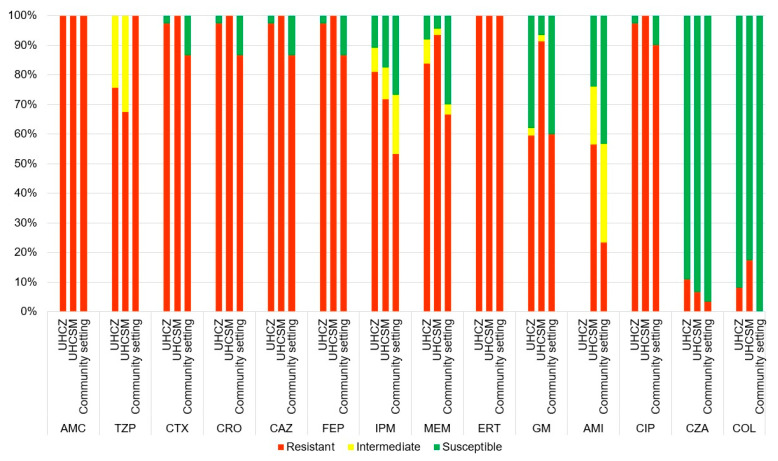
Comparison of antibiotic susceptibility between hospital and community setting isolates. Resistant, red; intermediate, yellow, susceptible, green. Abbreviations: AMC, amoxycilin–clavulanic acid; TZP, piperacillin–tazobactam;CAZ, ceftazidime; CTX, cefotaxime; CRO, ceftriaxone; ERT, ertapenem; FEP, cefepime; IPM, imipenem; MEM, meropenem; CZA, ceftazidime-avibactam; GM, gentamicin; CIP, ciprofloxacin; COL, colistin; R, resistant, UHCZ, University Hospital Centre Zagreb; UHCSM, University Hospital Centre Sestre Milosrdnice; AMI, amikacin.

Resistance to amoxicillin–clavulanate and ertapenem was confirmed in all 113 strains. Very high resistance rates (95.6%, *n* = 108) were observed for cefuroxime, ESC, and cefepime, with MIC values exceeding 128 mg/L. Aside from ESC, resistance rates were also high for ciprofloxacin (92.9%, *n* = 105), piperacillin–tazobactam (86.7%, *n* = 98), and gentamicin (71.7%, *n* = 81), as shown in Figure 2 and Table 1. The best activity was exerted by ceftazidime–avibactam, colistin, and amikacin, with 90.2% (*n* = 102), 91.1% (*n* = 103), and 61.9% (*n* = 70) of the isolates being susceptible (Table 1 and Figure 2; Supplementary Materials). Carbapenems exhibited heterogeneous susceptibility patterns, with ertapenem being resistant against all isolates and imipenem and meropenem being susceptible and intermediate susceptible (susceptible at increased exposure); (Supplementary Materials). Meropenem exerted higher resistance rates compared to imipenem (81.4% vs. 68.1%), as illustrated in Table 1. Intermediate susceptibility, or susceptibility at increased exposure, was observed predominantly with piperacillin–tazobactam (20.3%), imipenem (12.4%), and meropenem (4.4%), as shown in Figure 2. Resistance to cefiderocol was not reported. All three groups of isolates showed similar resistant profiles, as shown in Table 1; however, resistance rates to ESC were higher among hospital isolates and exceeded 97%, compared to community isolates with a resistance rate of 86.7% (Table 1). According to the resistance pattern to ESC, all isolates belonged to group I. The resistance rates to aminoglycosides and fluoroquinolones were also markedly lower in the isolates acquired in the community setting compared to the hospital isolates. The MIC_90_ of all cephalosporins, gentamicin, and ciprofloxacin exceeded 128 µg/mL. The most efficient antibiotics were imipenem and colistin, with an MIC_90_ of 32 µg/mL.

The DDST and combined disk test with clavulanic acid confirmed ESBL production in 90.3% (*n* = 102) of the isolates, whereas AmpC detection yielded negative results in all strains. ESBLs were more prevalent among hospital isolates, as shown in Table 1 (91.9% and 93.5%), compared to the community setting, in which the rate of ESBL positivity was 83.3%.

Using the chi-square test of independence, no statistically significant difference was detected when comparing hospital and community settings in terms of the prevalence of ESBL-positive strains (92.8% vs. 83.3%, *p* = 0.14).

Modified Hodge and CIM test had moderate sensitivity and detected 81.4% (*n* = 92) and 85.8% (*n* = 97) of the isolates positive for carbapenemase in the OKNV test (Supplementary Materials). The rates of Hodge and CIM positivity were higher in the community setting compared to the hospital isolates (100.0% vs. 73.0–76.1% and 100.0% vs. 76.1–86.4%, respectively). MHT successfully identified KPC-producing organisms but failed to detect carbapenemase activity in two out of seven NDM-producing strains (28%) and 19 OXA-48-producing strains (18%). The inhibitor-based test with EDTA revealed positive results, with all seven strains positive for NDM in OKNV. However, false-positive results were observed with 21.2% (*n* = 24) of the isolates producing OXA-48 and exhibiting a high level of resistance to carbapenems. The false-positive enlargement of the inhibition zone was seen only with meropenem (Supplementary Materials).

### 3.3. Molecular Detection of Resistance Genes

**Figure 3:**
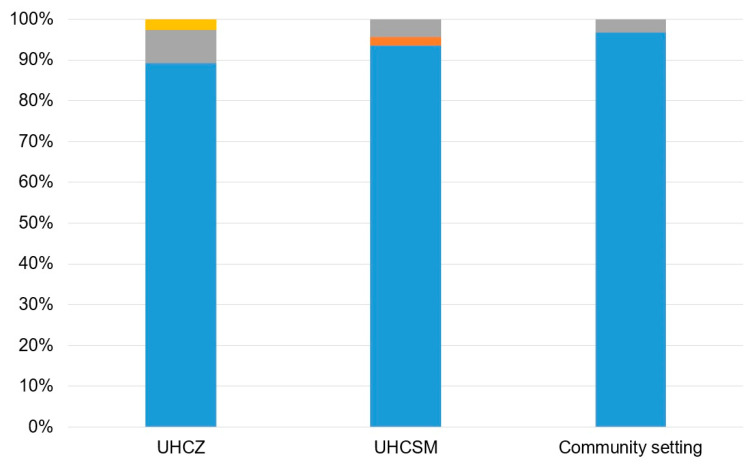
Distribution of carbapenemases in bacterial isolates according to the sampling location. OXA-48, blue; KPC, orange; NDM, gray; OXA-48+NDM, yellow.

A complete concordance was reported between routine immunochromatographic OKNV testing and PCR, which both detected OXA-48 in 93.8% (*n* = 106), NDM in 6.2% (*n* = 7), and KPC in only one isolate (0.9%). Double carbapenemases (OXA-48+NDM) were identified in one (0.9%) isolate, as illustrated in Figure 3.

Following confirmation by multiplex PCR, all ESBL-positive isolates (*n* = 102) were found to possess CTX-M β-lactamases belonging to cluster 1, as illustrated in Table 1 and Supplementary Materials. *bla*_TEM_ genes tested positive in nine hospital isolates, as shown in Table 1, and all generated TEM-1 allelic variants, while *bla*_SHV_ genes were found, as expected, in all isolates. *bla*_CTX-M_ genes in 25 isolates were linked to an upstream IS*Ecp1*-like element. PCR for *mcr* genes gave negative results in all ten colistin-resistant isolates.

The PCR mapping and sequencing of the flanking regions of the *bla*_OXA-48_ genes revealed the original structure reported by Gianni in 56 isolates [53]. Isolates positive for IS*1999* displayed elevated MICs of carbapenems and had positive phenotypic tests, proving carbapenem hydrolysis; however, some isolates resistant to carbapenems tested negative for insertion sequence, as shown in Supplementary Materials.

### 3.4. Detection of Resistance Genes by Inter-Array Kit CarbaResist

Three OXA-48-producing organisms, harbored almost identical resistance genes, as shown in Table 2. All possessed *bla_CTX-M-15_*, *bla_SHV_*, and *bla_OXA-1_* in addition to *bla_OXA-48_* genes. Resistance genes to non-β-lactam antibiotics were also identical: *aac(6′)-Ib* for aminoglycoside resistance, *dfrA14* for trimethoprim resistance, and *oqxA-* and *oqxB*-encoding efflux pumps. NDM-producing organism tested positive for *bla_CTX-M-15_*, *bla_SHV_*, *bla_OXA-1_*, and *bla_NDM_* genes, as well as *aac(6′)-Ib*, *dfrA14*, and *oqxA* and *oqxB*; additionally, it harbored *sul1* for sulphonamide resistance and *aphA*-encoding aminoglycoside resistance.

### 3.5. Transfer of Resistance Determinants

Twenty-one OXA-48-positive isolates conjugated successfully at a frequency of 10^−5^ to 10^−6^ (transconjugant/donor). Other resistance determinants were not cotransferred. Attempts to transfer cefotaxime resistance were unsuccessful. Carbapenem resistance levels were significantly lower in the transconjugants in comparison to the donors. PCR performed on transconjugant clones showed that no ESBL-resistant determinants had cotransferred alongside carbapenem-resistant genes.

### 3.6. Plasmid Characterization

**Figure 4:**
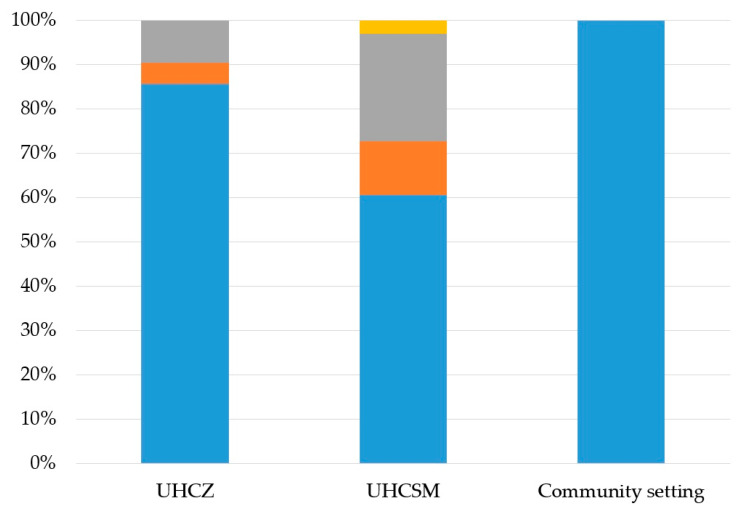
Distribution of plasmids in bacterial isolates according to the sampling location. IncL plasmid, blue; IncY plasmidorange; IncW plasmid, gray; IncP plasmid, yellow.

PBRT showed that the *bl_a__NDM_* gene was associated with the IncX plasmid in 3 isolates, and *bla*_OXA-48_ with the IncL plasmid in 48 isolates, as shown in Figure 4. IncW, IncY, and IncP occurred only sporadically in only 10, 5, and 1 isolate, respectively (Figure 4). No typable plasmids could be found in the 30 clinical isolates.

### 3.7. Genotyping

One isolate (35) was non-typable (gap-4, pho-4, pgi-57, inf-1, tonB-7, rpoB-1, and mdh-2), whereas the other (50) belonged to ST4531 (gap-2, pho-1, pgi-5, inf-6, tonB-6, rpoB-1, and mdh-2).

## 4. Discussion

Performing this study in two major medical centers in Croatia was a necessity to elucidate molecular mechanisms of resistance underlying the spread of carbapenemases among *K. pneumoniae* in Croatian hospitals and community settings in the recent turbulent years.

### 4.1. Carbapenemases

The main finding of the study is that OXA-48 combined with an ESBL belonging to the CTX-M family presented the most important contributor of ertapenem resistance among both hospital and community isolates and markedly outnumbered all other carbapenemases encountered in the past. However, in all except one strain, OXA-48-producing organisms were found to possess an additional ESBL, whereas OXA-48- and ESBL-negative strains were identified predominantly in the community setting. ESBL negativity among hospital isolates was linked to KPC and NDM carbapenemases, with only one ESBL-negative OXA-48-producing isolate originating from the hospital. Interestingly, VIM-MBLs and KPC were not found or found only sporadically in the case of KPC, and without any epidemiological link with international travel and therefore could be associated with the Balkan clone [58]. The dominance of OXA-48, mainly accompanied by an ESBL, has also been reported in other EU countries such as Germany, Romania, Slovenia, Bosnia and Herzegovina, France, Hungary, and Turkey [10,11,12,13,14,15,16,59,60].

In our neighboring country Slovenia, OXA-48 carried by incL/M plasmid is associated with nosocomial outbreaks, while NDM encoded on IncA/C plasmid occurred sporadically [15]. OXA-48 was also associated with monoclonal hospital outbreaks in Italy, which is on the other side of the Adriatic Sea [59]. The most frequent ST in these countries is ST11, which was never identified in Croatia. Both neighboring countries have intensive tourist and workforce exchange with Croatia [51,59]. A similar situation with the predominance of OXA-48 and sporadic occurrence of KPC and NDM was reported from countries at the eastern border of Croatia, namely Serbia [61] and Bosnia and Herzegovina [16], which have very intensive connections and population exchange with Croatia.

Hodge and CIM tests confirmed carbapenemase production in the majority of isolates identified as carbapenemase-positive in the routine OKNV test, although phenotypic tests missed some OXA-48 producers. CIM test exhibited better sensitivity, which is in concordance with reports from other authors [62]. The lower sensitivity of MHT in detecting MBLs, compared to other carbapenemases, is in line with other studies [62]. The resistance phenotype to imipenem and meropenem was inconsistent, ranging from complete susceptibility to plain resistance. On the other hand, uniform resistance to ertapenem was reported. This could be attributed to the overproduction of an additional ESBL found in virtually all isolates, combined with porin loss, which usually affects only ertapenem. False-positive results of the inhibitor-based test with EDTA were observed with isolates producing OXA-48, with a high level of resistance to carbapenems and only with the meropenem disk. The possible explanation is that EDTA converts oxacillinase to a less active state, leading to the enlargement of the inhibition zone around the meropenem disk.

### 4.2. Extended-Spectrum β-Lactamases

ESBLs belonging to the CTX-M family were identified as additional β-lactamases in isolates harboring OXA-48 and NDM, similar to the findings of Romanian and German studies [13,14]. Typically, CTX-M β-lactamases are known for their ability to rapidly hydrolyze cefotaxime, yet ceftazidime MICs exceeded 128 mg/L in the majority of the isolates. The explanation is that *bla_C__TX-M_* genes in all isolates belonged to cluster 1, with CTX-M-15 as the only allelic variant detected. This widespread variant provides the producing isolates with high-level resistance to all ESC and cefepime, particularly if linked to IS*Ecp*. Insertion sequence not only increased the level of resistance to ESC but also mediated the mobilization of the gene. However, conjugation experiments failed to transfer cefotaxime resistance, which indicated possible chromosomal incorporation of the genes. The rate of additional ESBL positivity, which conferred resistance to ESC, was much higher among hospital isolates compared to the community ones. Since the majority of isolates produced OXA-48, which does not hydrolyze cephalosporins, and all tested negative for p-AmpCs, resistance to ESC was conferred by an additional ESBL. Additional TEM-1 β-lactamase in some isolates may have contributed to resistance to β-lactam inhibitor combinations, although inhibitor resistance is typically linked to OXA-48. KPC-producing organism tested negative for an ESBL, which is in contrast to reports from other authors identifying TEM-116 or SHV-12 in addition to KPC-2 [63,64]. The presence of an additional ESBL has little effect on resistance phenotype, as KPC carbapenemases efficiently hydrolyze cephalosporins and render them resistant.

### 4.3. Plasmid-Mediated Amp-C β-Lactamases

P-AmpCs were not found among our isolates. The possible explanation is that cephamycins are not licensed for clinical use in Croatia, and thus there is no selection pressure to drive the spread of P-ampC genes. P-AmpCs belonging to the CMY family, namely CMY-4 and CMY-16 conferring resistance to cephamycins, were found in CRKP (NDM+OXA-48) in Bulgaria [65] and Switzerland [66]. The CMY-16-positive isolate coharbored CTX-M-15 and fluoroquinolone-resistant genes (*qnr*A and *arm*A) in addition to double carbapenemases. CMY-6 was present in an NDM-harboring isolate from Albania [67].

### 4.4. Other Resistance Genes

*qnr* genes were not found in fluoroquinolone-resistant isolates, indicating that fluoroquinolone resistance was most likely due to the mutations of chromosomal *gyr*A and *par*C genes, but the clarification of these resistance traits was beyond this study. *qnr*B genes were responsible for fluoroquinolone resistance in NDM-1 harboring *K. pneumoniae* from Bulgaria [65], whereas *qnr*A and *qnr*S were described in OXA-48-producing strains from Turkey [68], with *qnr*A showing the highest frequency, while *qnr*S conferred the highest level of quinolone resistance.

PCR for plasmid-mediated *mcr* genes was negative in all seven colistin-resistant strains, and therefore the resistance was probably attributed to the disruption of the *mgr*B gene by insertion sequence, as described previously in Italy [29]. Colistin-resistant ST 258 isolates were causative agents of an outbreak in Italy, spreading in different hospital wards [31]. In the present study, we only excluded plasmid-encoded, transferable resistance. Resistance associated with *mcr* genes encoded on transferable genetic elements has mostly been reported from China, at the beginning among animal isolates and later in clinical settings [69].

### 4.5. Antibiotic Susceptibility

OXA-48-producing isolates exhibited variable MICs of imipenem and meropenem, with a significant proportion of isolates being susceptible. Only ertapenem demonstrated uniform resistance. Resistance to imipenem and meropenem was related to the presence of the IS*1999* insertion sequence, which is in concordance with its role as a promotor driving the expression of the gene and the level of carbapenem resistance. The isolates without detected insertion elements showed MICs of imipenem and meropenem in the susceptible or intermediate-susceptible range. OXA-48-producing isolates displayed uniform resistance to amoxicillin–clavulanate, which is in concordance with the substrate profile and inhibitor sensitivity of OXA-48. However, piperacillin–tazobactam had a significant proportion of isolates in the intermediate or increased-exposure category, although OXA-48 harbored by the majority of the strains confers high-level resistance to all β-lactam inhibitor combinations. Cumulative MIC values and the rate of resistant isolates were higher for meropenem in spite of the fact that oxacillinases preferentially hydrolyze imipenem. This could be attributed to additional resistance mechanisms such as the hyperexpression of efflux systems, which affect meropenem more than imipenem. MBLs harbored by the minority of the isolates pose a serious therapeutic problem because they hydrolyze all β-lactam antibiotics except aztreonam, which is not available in Croatia, and they evade all approved β-lactamase inhibitors, leaving only a few antibiotics active. The majority of the isolates were resistant to almost all β-lactam antibiotics. The high rate of strains intermediate susceptible to imipenem and meropenem is in line with the predominance of OXA-48 carbapenemase with typical variable susceptibility to carbapenems.

Cefiderocol showed the best activity, with no resistant isolates observed. Resistance to ceftazidime–avibactam was linked to MBL production. The implementation of the immunochromatographic OKNV test [45], as a very useful tool for the rapid identification of the carbapenemase class, enables clinical microbiologists to provide recommendations for therapy before susceptibility testing is finished. Ceftazidime–avibactam is recommended if OXA-48 is confirmed, while novel inhibitor combinations are ruled out if MBL is identified.

### 4.6. Plasmid Characterization

OXA-48 was carried by the L-type plasmid, reinforcing the hypothesis that the current spread of OXA-48-carrying genes is mainly the consequence of the diffusion of an epidemic plasmid. Previous studies have shown that plasmids carrying *bla_OXA-48_* genes share similar features in that they have an incL/M backbone and are very similar in size (60–70 kb), suggesting their wide dissemination in different species and countries via an epidemic plasmid [10,11,12,13,14,15]. This plasmid was previously shown to play a major role in the diffusion of OXA-48-encoding genes. IncP, IncFIC, and IncW plasmids were found only sporadically and are known to carry ESBL-encoding genes. The IncX plasmid identified among NDM-producing organisms was found among NDM-positive hypervirulent strains in Japan [70]; however, in most studies, IncA/C and IncF plasmids harbor NDM-encoding genes [71,72].

### 4.7. Genotyping

ST4531, identified in one isolate, was not reported before. The majority of OXA-48-producing isolates in other EU countries belong to ST395 and ST11 [13,14,15,59], while ST258 usually carries KPC-encoding genes [20,21,22,23,24]. A limitation of this study is that genotyping was not performed on all isolates to determine the clonal lineages carrying certain carbapenemase-encoding genes.

## 5. Conclusions

This study proved the predominance of OXA-48 carbapenemase, driven by L plasmids among CRKP in Croatia, encompassing not only hospital but also community settings. There was no significant difference in the prevalence of certain carbapenemase types, ESBL content, antibiotic susceptibility, and plasmid profiles between the three population groups, reinforcing the “one health” theory.

The results presented in the study warrant the development of practical containment methods alongside strong and immediate interventions in both major medical centers to prevent the spread of resistant bacteria. Carbapenem resistance in *K. pneumoniae* is a global phenomenon, and it is necessary to monitor the resistance traits of this important hospital pathogen and analyze the mobile genetic elements responsible for the rapid diffusion of its resistance traits. In Croatia, a member of the European Union, a national surveillance system exists that monitors carbapenemases in Gram-negative bacteria in order to effectively combat the emerging threat.

The most important message of this study to take home is the long-lasting dominance of OXA-48 carbapenemase among CRKP in Croatia changing the face of carbapenemases in Croatia. The accumulation of resistance determinants underscores the necessity for stringent infection control measures and robust antimicrobial stewardship programs to curb MDR bacteria.

## Figures and Tables

**Table 1:** Comparison of antibiotic susceptibility, β-lactamase production, and plasmid incompatibility groups of *K. pneumoniae* isolates between hospital and community isolates.

Characteristics	UHCZ	UHCSM	Community Isolates
Number of isolates	37	46	30
Number and % of ESBL-positive isolates	91.9% (34/37)	93.5% (43/46)	83.3% (25/30)
Number and % of AmpC-positive isolates	0.0% (0/37)	0.0% (0/46)	0.0% (0/30)
HODGE test	73.0% (27/37)	76.1% (35/46)	100.0% (30/30)
CIM test	86.4% (32/37)	76.1% (35/46)	100.0% (30/30)
AMC-R	100.0% (37/37)	100.0% (46/46)	100.0% (30/30)
TZP-R	100.0% (37/37)	67.4% (31/46)	100.0% (30/30)
CXM-R	97.3% (36/37)	100.0% (46/46)	86.7% (26/30)
CAZ-R	97.3% (36/37)	100.0% (46/46)	86.7% (26/30)
CTX-R	97.3% (36/37)	100.0% (46/46)	86.7% (26/30)
CRO-R	97.3% (36/37)	100.0% (46/46)	86.7% (26/30)
FEP-R	97.3% (36/37)	100.0% (46/46)	86.7% (26/30)
IMP-R	75.7% (28/37)	71.7% (33/46)	53.3% (16/30)
MEM-R	78.4% (29/37)	93.5% (43/46)	66.7% (20/30)
ERT-R	100.0% (37/37)	100.0% (46/46)	100.0% (30/30)
GM-R	62.1% (23/37)	89.1% (41/46)	56.7% (17/30)
AMI-R	32.4% (12/37)	56.5% (26/46)	16.7% (5/30)
CIP-R	94.6% (35/37)	100.0% (46/46)	80.0% (24/30)
COL-R	8.1% (3/37)	15.2% (7/46)	0.0% (0/30)
CZA-R	8.1% (3/37)	17.4% (8/46)	0.0% (0/30)
*bla*_CTX-M_	91.9% (34/37)	93.4% (43/46)	83.3% (25/30)
*bla*_TEM_	5.4% (2/37)	15.2% (7/46)	0.0% (0/30)
*bla*_OXA-48_	91.9% (34/37)	93.4% (43/46)	96.7% (29/30)
*bla*_KPC_	0.0% (0/37)	2.2% (1/46)	0.0% (0/30)
*bla*_NDM_	10.8% (4/37)	4.3% (2/46)	3.3% (1/30)
Inc L plasmid	48.6% (18/37)	43.5% (20/46)	33.3% (10/30)
IncY plasmid	2.7% (1/37)	8.7% (4/46)	0.0% (0/30)
IncW plasmid	5.4% (2/37)	17.4% (8/46)	0.0% (0/30)
IncP plasmid	0.0% (0/37)	2.2% (1/46)	0.0% (0/30)

Abbreviations: AMC, amoxycilin–clavulanic acid; TZP, piperacillin–tazobactam; CXM, cefuroxime; CAZ, ceftazidime; CTX, cefotaxime; CRO, ceftriaxone; FEP, cefepime; ERT, ertapenem; IPM, imipenem; MEM, meropenem; GM, gentamicin; AMI, amikacin; CIP, ciprofloxacin; COL, colistin; CZA, ceftazidime–avibactam; R, resistance, UHCZ, University Hospital Centre Zagreb; UHCSM, University Hospital Centre Sestre Milosrdnice; R, resistance; CIM, carbapenem inactivation method.

**Table 2:** Inter-array chip results of four *K. pneumoniae* isolates.

Isolate and Protocol Number	Center	β-Lactam	AG	SUL	THR	Efflux
*K. pneumoniae* 36 (47168)	UHCSM	*bla* *_CTX-M-15_* *bla* *_SHV_* *bla* *_OXA-1_* *bla* *_OXA-48_*	*aac(6*′*)-Ib*		*dfrA14*	*oqxA*, *oqxB*
*K. pneumoniae* 38 (39118)	UHCSM	*bla* *_CTX-M-15_* *bla* *_SHV_* *bla* *_0XA-48_*	*aac(6*′*)-Ib*		*dfrA14*	*oqxA*, *oqxB*
*K. pneumoniae* 39 (23199)	UHCSM	*bla* *_CTX-M-15_* *bla* *_SHV_* *bla* *_OXA-1_* *bla* *_OXA-48_*	*aac(6*′*)-Ib*		*dfrA14*	*oqxA*, *oqxB*
*K. pneumoniae* 40 (152854)	UHCZ	*bla* *_CTX-M-15_* *bla* *_SHV_* *bla* *_OXA-1_* *bla* *_NDM_*	*aac(6*′*)-Ib* *aphA*	*sul1*		*oqxA*,

Abbreviations: UHCZ, University Hospital Centre Zagreb; UHCSM, University Hospital Centre Sestre Milosrdnice; AG, aminoglycosides; SUL, sulphonamides; THR, trimethoprim.

## Data Availability

The original contributions presented in the study are included in the article/Supplementary Material, further inquiries can be directed to the corresponding author.

## Supplementary Materials

The following supporting information can be downloaded at: https://www.mdpi.com/article/10.3390/microorganisms12112224/s1.
